# Quantification of functional weakness and abnormal synergy patterns in the lower limb of individuals with chronic stroke

**DOI:** 10.1186/1743-0003-3-17

**Published:** 2006-07-20

**Authors:** Nathan Neckel, Marlena Pelliccio, Diane Nichols, Joseph Hidler

**Affiliations:** 1 Center for Applied Biomechanics and Rehabilitation Research(CABRR), National Rehabilitation Hospital, 102 Irving Street, NW, Washington, DC 20010, USA; 2 Physical Therapy Service, National Rehabilitation Hospital, 102 Irving Street, NW, Washington, DC 20010, USA; 3 Department of Biomedical Engineering, Catholic University, 620 Michigan Ave., NE, Washington, DC 20064, USA

## Abstract

**Background:**

The presence of abnormal muscle activation patterns is a well documented factor limiting the motor rehabilitation of patients following stroke. These abnormal muscle activation patterns, or synergies, have previously been quantified in the upper limbs. Presented here are the lower limb joint torque patterns measured in a standing position of sixteen chronic hemiparetic stroke subjects and sixteen age matched controls used to examine differences in strength and coordination between the two groups.

**Methods:**

With the trunk stabilized, stroke subjects stood on their unaffected leg while their affected foot was attached to a 6-degree of freedom load cell (JR3, Woodland CA) which recorded forces and torques. The subjects were asked to generate a maximum torque about a given joint (hip abduction/adduction; hip, knee, and ankle flexion/extension) and provided feedback of the torque they generated for that primary joint axis. In parallel, EMG data from eight muscle groups were recorded, and secondary torques generated about the adjacent joints were calculated. Differences in mean primary torque, secondary torque, and EMG data were compared using a single factor ANOVA.

**Results:**

The stroke group was significantly weaker in six of the eight directions tested. Analysis of the secondary torques showed that the control and stroke subjects used similar strategies to generate maximum torques during seven of the eight joint movements tested. The only time a different strategy was used was during maximal hip abduction exertions where stroke subjects tended to flex instead of extend their hip, which was consistent with the classically defined "flexion synergy." The EMG data of the stroke group was different than the control group in that there was a strong presence of co-contraction of antagonistic muscle groups, especially during ankle flexion and ankle and knee extension.

**Conclusion:**

The results of this study indicate that in a standing position stroke subjects are significantly weaker in their affected leg when compared to age-matched controls, yet showed little evidence of the classic lower-limb abnormal synergy patterns previously reported. The findings here suggest that the primary contributor to isometric lower limb motor deficits in chronic stroke subjects is weakness.

## Background

Muscle weakness, or the inability to generate normal levels of force, has clinically been recognized as one of the limiting factors in the motor rehabilitation of patients following stroke [1,2]. In the lower limbs, this muscle weakness can be attributed to disuse atrophy [3] and/or the disruption in descending neural pathways leading to inadequate recruitment of motorneuron pools [1,4-6]. It has also been reported that weakness following stroke may be the result of co-contraction of antagonistic muscles [7-9]. Spasticity has also been proposed as an alternative explanation for lower limb impairments in hemiparetic stroke [10,11], but more recent studies have found that spasticity may not play a significant role in gait abnormalities [12,13].

A well documented factor limiting the motor rehabilitation of patients following stroke is the presence of abnormal muscle activation patterns. Following stroke, some patients lose independent control over select muscle groups, resulting in coupled joint movements that are often inappropriate for the desired task [14,15]. These coupled movements are known as synergies and, for the lower limb, have been grouped into the extension synergy (internal rotation, adduction, and extension of the hip, extension of the knee and extension and inversion of the ankle) and the flexion synergy (external rotation, abduction, and flexion of the hip, flexion of the knee, and flexion and eversion of the ankle) [16,17] with varying levels of completeness [18] and dominance [19].

Much of the literature attempting to quantify these abnormal muscle synergies is focused on the paretic upper limb of stroke patients. In isometric conditions, it has been shown that stroke patients have a limited number of upper limb synergies available to them due to abnormal muscle coactivation patterns [20]. In dynamic tasks, abnormal synergy patterns exist in the paretic upper limb between shoulder abduction with elbow flexion as well as shoulder adduction with elbow extension [21]. These, and other inappropriate upper limb muscle synergy patterns were attributed to abnormal torque generation about joints secondary to the intended, or primary, joint axis during maximal voluntary isometric contractions [22].

This analysis technique of quantifying torques at joints secondary to the intended joint axis was applied to the lower limbs of cerebral palsy patients in a seated position, where abnormal secondary joint torques were expressed during maximal hip and knee extension [23]. However, it has been shown that gravity can influence the control of limb movements by affecting sensory input [24] and altering task mechanics [25,26]. When acute (<6 weeks post-injury) stroke subjects were placed in a functionally relevant weight-bearing anti-gravity standing position, no such abnormal secondary joint torque patterns during maximal voluntary isometric contractions were found, even though primary joint torques deficits were observed [27].

The goal of this study was to quantify lower limb weakness and coordination in chronic (> 1 year post-injury) stroke patients in a functionally relevant standing position. Subjects were asked to generate maximum isometric contractions about a given joint while torques at joints secondary to the desired exertion were simultaneously calculated and recorded. This allowed us to quantify weakness as a torque deficit and coordination as the generation of any synergy patterns in the lower limbs of hemiparetic stroke patients. Additionally, EMG activity of relevant muscles was simultaneously recorded to quantify the presence of abnormal muscle activation patterns.

## Methods

### Subjects

Sixteen subjects (9 male, 7 female) with hemiparesis resulting from a single unilateral cortical or sub-cortical brain lesion at least one year prior to testing participated in this study along with sixteen (9 male, 7 female) neurologically intact age-matched controls. Subjects were excluded from the study if they were too severely impaired to voluntarily move about the ankle, knee, and hip joints, measured by a Fugl-Meyer lower limb score below 10 out of 34. Subjects with a Fugl-Meyer lower limb score greater than 30 out of 34 were deemed very highly functional and excluded. The synergy control sub-score of the Fugl-Meyer assessment was also used to characterize subjects. This clinical score (0–22) reflects the ability to move within (0–14), to combine (15–18), or to move out of (19–22) classically defined dynamic synergy patterns. Although some subjects scored high on the Fugl-Meyer lower limb and synergy control sub-score, all subjects exhibited difficulty in walking typical of hemiplegic stroke subjects. Subjects were also screened for cognitive and communication impairments and only those with Mini Mental State Examination scores greater or equal to 22 were tested. All subjects were excluded for any uncontrolled cardiovascular, neurological, or orthopaedic conditions, such as high blood pressure, arthritis, or history of seizure, that would inhibit exercise in a standing position. Informed consent was obtained before testing and all protocols were approved by the local institutional review boards. The clinical characteristics of each subject group is shown in Table 1.

**Table 1:** Clinical Characteristics of Subjects

**Group**	**Gender**	**Age (years)**	**Paretic Leg Tested**	**Months Post-Stroke**	**Synergy Control (max. = 22)**	**Fugl-Meyer Score %**
Stroke Survivors	F	30	R	39	13	79
	F	36	R	26	21	88
	F	48	R	13	21	68
	F	51	L	54	21	91
	F	53	L	36	6	53
	F	57	L	26	20	53
	F	64	R	14.5	9	88
	M	44	R	149	17	71
	M	50	R	194	16	53
	M	50	R	29	14	56
	M	55	R	34	10	68
	M	56	R	30	15	47
	M	59	L	13.5	16	44
	M	63	L	23	11	47
	M	68	L	20	19	47
	M	69	R	18.5	11	76

Stroke	9 male	53.31	10 right leg	44.97	15	64.34
Average	7 female	(+/-10.68)	6 left leg	(51.18)	(4.72)	(16.46)

Control Average	9 male 7 female	57.13 (+/-8.85)	10 right leg 6 left leg	/	/	/

Standard Deviation in parenthesis

### Instrumentation

**Figure 1:**
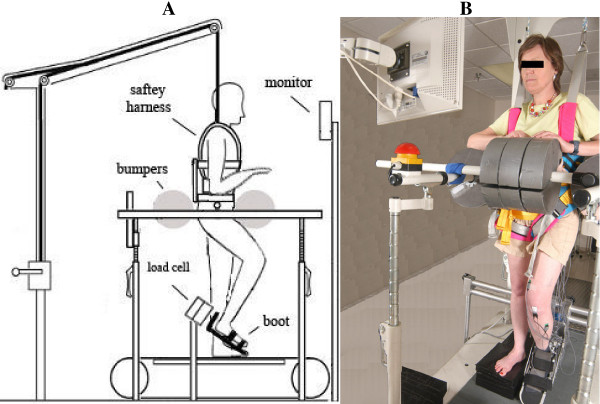
**Experimental Set-up**. **A. **Subjects were secured in a standing position with foam bumpers pinching the hips from four sides and a safety harness prevented subjects from slipping down. The subject's foot was attached to a boot that was fixed to a six DOF load cell that would measure joint torques about the hip, knee and ankle. A monitor provided feedback on the torque generated in the primary joint direction. EMG activity was recorded from eight muscles. **B**. Photograph of experimental setup.

Each subject was placed in a custom setup that allowed for the study of strength and coordination of the lower extremities in a standing posture (Figure 1). The subject's affected foot was securely placed inside a custom foot retainer which in turn was connected to a 6-axis load cell (JR3, Woodland CA). The foot retainer was angled down 30 degrees with respect to the horizontal so that all subjects had an ankle angle of 100 degrees and a knee angle of 135 degrees. Large foam bumpers were used to support the subject's trunk during the exertions. Because the tests were done with the subject in a standing posture, a harness was placed around the subject's abdomen and attached to an over-head body-weight support system in order to prevent falls. No support was provided by the system during the tests. Some subjects did, however, sit down in the harness between trials to rest their support leg. Additionally, a heart rate monitor was placed around the subject's chest which was repeatedly checked during testing by a physical therapist to ensure the exertions did not elevate the subject's heart rate to unsafe levels. A monitor for biofeedback was placed in front of the subjects to reinforce exertions along each joint axis.

Electromyographic (EMG) recordings were collected using a Bagnoli-8 EMG system (Delsys, Inc., Boston, MA) with surface electrodes placed above the muscle belly's of the tibilias anterior, gastrocnemius, biceps femoris, vastus medialis, rectus femoris, gluteus maximus, gluteus medius, and adductor longus, and a common reference electrode placed on the patella. Electrode sites were abraded with a rough sponge and cleaned with isopropyl alcohol. The Ag-AgCl electrodes (contact dimension 10 mm × 1 mm, contact spacing 10 mm) were prepped with adhesive stickers and electrode gel. The preamplifiers provided a gain of ×10+-2%, the amplifiers a gain selectable from ×100 to ×10,000 with a bandwidth of 20–450 Hz. The common mode rejection ratio was >80 dB at 60 Hz and the input impedance was >10^15^//0.2 ohm//pF.

EMG data, along with the forces and torques from the load cell, were anti-alias filtered at 500 Hz prior to sampling at 1000 Hz using a 16-bit data acquisition board (Measurement Computing, PCI-DAS 6402, Middleboro, MA) and custom data acquisition software written in Matlab (Mathworks Inc. Natick, MA) and stored for later analysis.

### Protocol

Subjects were asked to generate maximum voluntary torques (MVTs) about eight different joint directions (ankle, knee, and hip flexion and extension, as well as hip abduction and adduction). For each joint direction, the subject was allowed to practice until they understood the task, after which three trials were recorded. Subjects were watched closely to make sure that they maintained their legs in the proper geometry. Trials were discarded and re-collected if subjects attempted to change leg geometry in order to achieve maximum torques. A minimum of one minute rest period was given between each trial. The subjects would start in a relaxed state and slowly ramp up to a maximum which was held for approximately 4 seconds. Visual feedback of the torque generated only along the desired direction was provided by a speedometer style display on the monitor. The order of joint movements was selected to minimize subject fatigue (hip adduction, knee flexion, hip extension, ankle flexion, hip abduction, knee extension, hip flexion, ankle extension). All subjects followed the same order of selected joint torques. Verbal encouragement and instructions were provided throughout the experiment.

### Data analysis

For each trial the MVT, or primary torque, as well as the three secondary torques were measured along with the EMG data from the eight selected muscles. The different joint torques were computed by taking the forces and torques measured by the load cell (denoted frame {o}) and transforming them back to the different joints using a homogeneous transformation matrix [28]. From the load cell, ankle torques can be calculated from:



where  is a 3 × 3 rotation matrix from {o} to {a},  is a 3 × 3 skew matrix from {o} to {a}, and F_i _and T_i _denote force and torque in each respective frame.

Ankle forces and moments can then be transformed back to the knee as:



And from the knee to hip as:



The skew and rotation matrices are formed from anatomical measurements while the subject is in the setup (shank and thigh lengths, knee and shank angles).

A MVT was defined as the peak torque sustained for 200 ms observed across any one of the 3 trials for that primary joint direction. The corresponding secondary torques exerted along the other joint axes during the 200 ms MVT window were also identified. For example, during maximum voluntary knee flexion exertions, secondary torques consisted of those generated along the ankle flexion-extension axis, hip flexion-extension axis, and hip abduction-adduction axis. Secondary torques generated during all trials were normalized to the MVT measured for that particular joint direction. Cases where a secondary torque exceeded 100% MVT indicated that the subject generated less torque while attempting to maximize that particular direction than when they were trying to maximize a different direction.

The EMG activity from the eight selected muscle groups was band-pass filtered (20–450 Hz), full-wave rectified, and then smoothed using a 200-point RMS algorithm. Each EMG trace was then normalized to the maximum EMG value observed across all trials for the respective muscle. This allowed for muscle activity demonstrated during the 200 ms MVT window to be expressed as the percentage of peak activity observed in each muscle.

### Statistical analysis

A single factor ANOVA was used to compare the means of the chronic stroke subjects to the control subjects for each of the eight primary joint torque directions. A single factor ANOVA was used to compare the mean secondary torques, as well as the mean EMGs, between the stroke and control groups. An independent Student's *t*-test was used to identify secondary torques that were significantly greater than zero (P < 0.05). Correlations (Pearson's, 2-tailed) between joint torque were found by grouping all data from the eight primary torque directions and comparing all instances of one torque direction with the activity at the other three joints. For example, all instances of hip abduction were compared with the torques of the hip, knee and ankle, regardless if it was flexion or extension. Statistical analyses was performed with the software package SPSS (SPSS Inc, Chicago, IL) and a confidence level of 0.05 was used for all comparisons.

The role of co-activation of antagonistic muscles on observed joint weakness was investigated by computing a co-contraction index (*CI*) for each primary torque direction as follows:



where *PCSA *is the physiological cross sectional area of the healthy adult muscle [29]. The total activity demonstrated in the agonist muscle groups divided by the total muscle activity demonstrated in the antagonistic muscle groups results in the CI for that primary torque direction. One or more of the eight muscles recorded from were regarded as agonist/antagonist muscles for each primary torque direction (ankle flexor – tibilias anterior, ankle extensor – gastrocnemius, knee flexors – gastrocnemius and biceps femoris, knee extensors – vastus medialis and rectus femoris, hip flexor – rectus femoris and adductor longus, hip extensors – gluteus maximus and biceps femoris, hip abductor – gluteus medius, hip adductor – adductor longus and gluteus maximus). It was important to scale the muscle activity by the PCSA since activity in large muscle groups generated significantly higher forces than activity in muscles with smaller cross-sectional area. The CI is a simple numerical measure of how much co-activation of antagonistic muscle groups subjects exhibit. Low CI occurs when subjects simultaneously activate agonist and antagonist muscle groups, whereas high CI is indicative of low levels of co-contraction. High levels of co-contraction (Low CI) would result in decreasing levels of torque exerted at the joint. A single factor ANOVA test was used to compare the mean CI values of the chronic stroke subjects to the control subjects with a significance level of p < 0.05.

## Results

### Maximum voluntary torque

**Figure 2:**
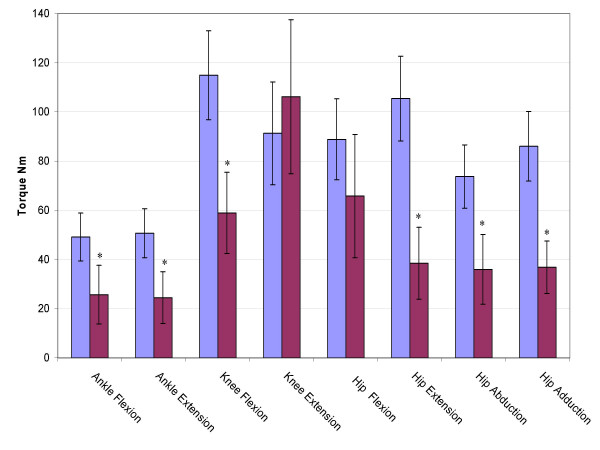
**Maximum Voluntary Torques**. The maximum voluntary joint torques for the stroke (red) and control (blue) groups expressed in Newton meters for the eight primary directions ankle flexion through hip adduction. Error bars represent 95% confidence interval. Significant differences (p < 0.05) are denoted *.

The maximum voluntary primary torques for the eight joint directions are shown in figure 2. The stroke group was significantly weaker (p < 0.05) for all joint directions except for knee extension and hip flexion. The average stroke hip flexion torque was less than the control group, but with a higher variability. The average stroke knee extension torque was actually larger than the control group, but again, with a higher variability.

### Secondary torque and EMG patterns

**Figure 3:**
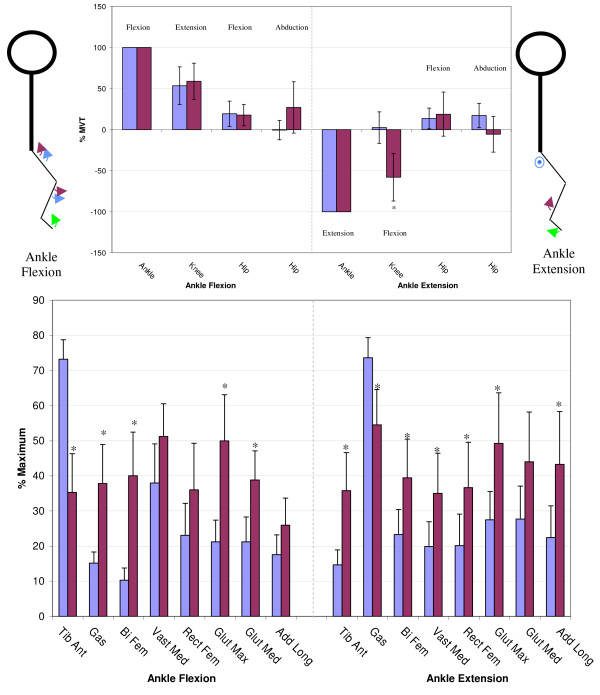
**Secondary Torques During Ankle Flexion/Extension**. The top graphs show the secondary joint torques for the stroke (red) and control (blue) groups expressed in %MVT for ankle flexion (left) and ankle extension (right). The stick figures show the primary joint direction (green) as well as the secondary torques of the control (blue) and stroke (red) for the secondary joint torques that are significantly greater than zero. Abduction is denoted as a circled dot (out of the page), adduction is denoted a circled X (into the page). The bottom graph shows the EMG activity for the stroke (red) and control (blue) groups expressed in % maximum value during ankle flexion MVT (left) and ankle extension MVT (right). Error bars represent 95% confidence interval. Significant differences between groups (p < 0.05) are denoted *. Tib Ant – tibilias anterior, Gas – gastrocnemius, Bi Fem – biceps femoris, Vast Med – vastus medialis, Rect Fem – rectus femoris, Glut Max -gluteus maximus, Glut Med – gluteus medius, Add Long – adductor longus.

Figures 3 through 6 show the normalized secondary torque patterns as well as the normalized EMG activity for all control subjects and all but one stroke subject during the eight different primary directions. EMG data for one stroke subject was improperly collected and has hence been omitted. The stick figure diagrams illustrate the secondary torque generation that was significantly greater than zero (P < 0.05). A more detailed discussion of the different joint directions is presented below.

**Figure 4:**
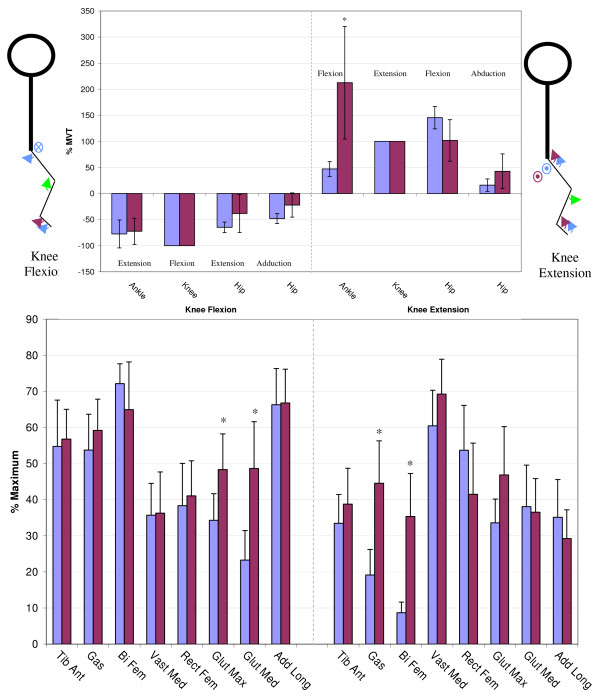
**Secondary Torques During Knee Flexion/Extension**. The top graphs show the secondary joint torques for the stroke (red) and control (blue) groups expressed in %MVT for knee flexion (left) and knee extension (right). The stick figures show the primary joint direction (green) as well as the secondary torques of the control (blue) and stroke (red) for the secondary joint torques that are significantly greater than zero. Abduction is denoted as a circled dot (out of the page), adduction is denoted a circled X (into the page). The bottom graph shows the EMG activity for the stroke (red) and control (blue) groups expressed in % maximum value during knee flexion MVT (left) and knee extension MVT (right). Error bars represent 95% confidence interval. Significant differences between groups (p < 0.05) are denoted *. Tib Ant – tibilias anterior, Gas – gastrocnemius, Bi Fem – biceps femoris, Vast Med – vastus medialis, Rect Fem – rectus femoris, Glut Max -gluteus maximus, Glut Med – gluteus medius, Add Long – adductor longus.

**Figure 5:**
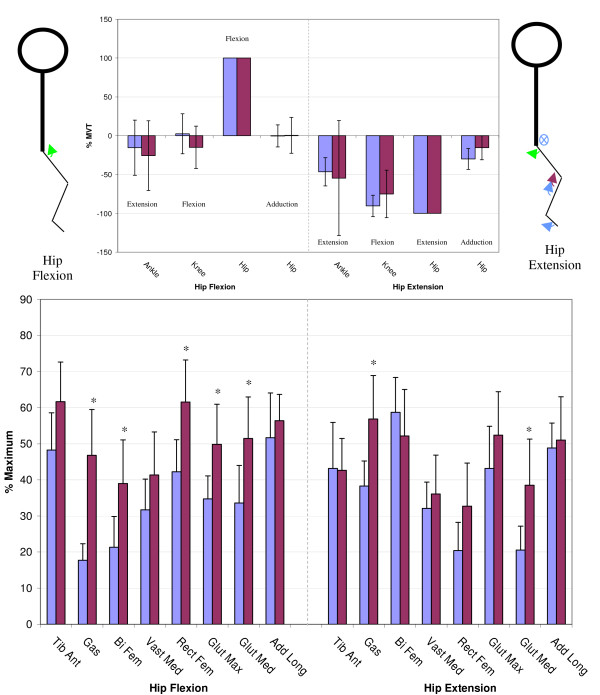
**Secondary Torques During Hip Flexion/Extension**. The top graphs show the secondary joint torques for the stroke (red) and control (blue) groups expressed in %MVT for hip flexion (left) and hip extension (right). The stick figures show the primary joint direction (green) as well as the secondary torques of the control (blue) and stroke (red) for the secondary joint torques that are significantly greater than zero. Abduction is denoted as a circled dot (out of the page), adduction is denoted a circled X (into the page). The bottom graph shows the EMG activity for the stroke (red) and control (blue) groups expressed in % maximum value during hip flexion MVT (left) and hip extension MVT (right). Error bars represent 95% confidence interval. Significant differences between groups (p < 0.05) are denoted *. Tib Ant – tibilias anterior, Gas – gastrocnemius, Bi Fem – biceps femoris, Vast Med – vastus medialis, Rect Fem – rectus femoris, Glut Max -gluteus maximus, Glut Med – gluteus medius, Add Long – adductor longus.

**Figure 6:**
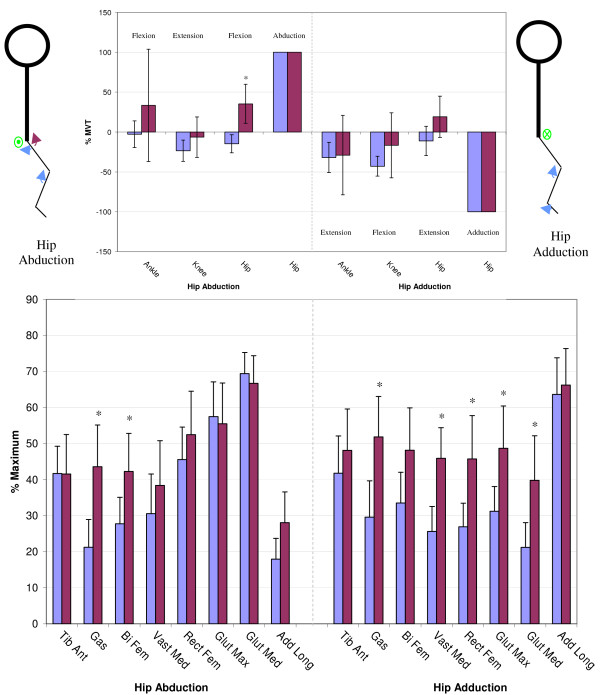
**Secondary Torques During Hip Abduction/Adduction**. The top graphs show the secondary joint torques for the stroke (red) and control (blue) groups expressed in %MVT for hip abduction (left) and hip adduction (right). The stick figures show the primary joint direction (green) as well as the secondary torques of the control (blue) and stroke (red) for the secondary joint torques that are significantly greater than zero. Abduction is denoted as a circled dot (out of the page), adduction is denoted a circled X (into the page). The bottom graph shows the EMG activity for the stroke (red) and control (blue) groups expressed in % maximum value during hip abduction MVT (left) and hip adduction MVT (right). Error bars represent 95% confidence interval. Significant differences between groups (p < 0.05) are denoted *. Tib Ant – tibilias anterior, Gas – gastrocnemius, Bi Fem – biceps femoris, Vast Med – vastus medialis, Rect Fem – rectus femoris, Glut Max -gluteus maximus, Glut Med – gluteus medius, Add Long – adductor longus.

#### Ankle flexion/extension

As illustrated in Figure 3, during ankle flexion, both controls and stroke subjects generated knee extension and hip flexion secondary torques. While generating maximal ankle flexion, the stroke subjects had significantly less tibilias anterior activity but significantly greater gastrocnemius, biceps femoris, gluteus maximus, and gluteus medius activity. During maximal ankle extension exertions, the stroke subjects generated a knee flexion secondary torque that was significantly higher than the control subjects (p < 0.05). The EMG pattern on the right side of figure 3 shows that the stroke subjects had significantly less gastrocnemius muscle activity and significantly greater tibilias anterior, biceps femoris, vastus medialis, rectus femoris, gluteus maximus, and adductor longus muscle activity during maximal ankle extension exertions.

#### Knee flexion/extension

During maximal knee flexion exertions, both groups generated ankle extension, hip extension and hip adduction secondary torques that were not different from each other (Figure 4). Interestingly, the stroke subjects had significantly greater gluteus maximus, and gluteus medius activity during maximum knee flexion exertions despite the fact that they did not produce larger hip extension secondary torque. For knee extension, both groups produced ankle flexion, hip flexion and hip abduction secondary torques however the ankle flexion secondary torque was significantly larger in the stroke group, and significantly greater than 100%. The hip flexion secondary torque was also greater than 100% in the control group but not significantly different than the stroke group. The EMG pattern illustrates that the stroke group had a greater gastrocnemius and biceps femoris activity during knee extension MVT.

#### Hip flexion/extension

Figure 5 illustrates the secondary torques generated during hip flexion, where it can be seen that neither group generated significant secondary torques. However the stroke group produced greater activity in the gastrocnemius, biceps femoris, rectus femoris, gluteus maximus, and gluteus medius muscles. During hip extension MVT, both groups produced a secondary knee flexion torque and the control group produced additional ankle extension and hip adduction secondary torques that were not significantly different from the stroke. The EMG pattern in figure 5 shows that the stroke group had greater gastrocnemius and gluteus medius activity during hip extension MVT.

#### Hip abduction/adduction

During hip abduction, the control group produced a hip extension secondary torque while the stroke group produced a hip flexion secondary torque, the difference being significantly different (Figure 6). During hip abduction MVT, the stroke subjects had significantly greater gastrocnemius and biceps femoris activity than the control subjects. For hip adduction MVT, none of the secondary torques were significantly different. The EMG pattern on the right side of figure 6 illustrates how the stroke group had greater gastrocnemius, vastus medialis, rectus femoris, gluteus maximus, and gluteus medius activity than the control subjects during hip adduction MVT.

#### Summary of secondary torques

For each group the secondary torques significantly greater than zero for the eight primary joint directions (figures 3 through 6) are summarized in Table 2. For each primary joint direction listed on the left, the secondary torques significantly greater than zero are marked with an 'X'. Additionally, significant correlations (p < 0.05) between joint torques within each group are marked with an 'O'. To find these correlations all instances (primary or secondary) of a torque were pooled and compared to the other three joint torques. For example, all trials where ankle flexion was present were pooled and ankle flexion was compared to knee flexion/extension, hip flexion/extension, and hip abduction/adduction. The arrangement of rows and columns in Table 2 leads to the grouping of the primary joint directions into synergies. These synergies are based on the direction of the moment arm of the joint torque in the sagittal plane. Ankle flexion, knee extension, and hip flexion secondary torques are grouped as the Anterior Synergy while ankle extension, knee flexion, and hip extension are grouped as the Posterior Synergy. The frontal plane joint torques of hip abduction and adduction are differently grouped. Hip adduction is part of the posterior synergy in the control group but not part of any synergy in the stroke group. Hip abduction is part of the anterior synergy in the stroke group but part of the posterior synergy in the control group.

**Table 2:** Secondary Torque Synergies

	Control	Ankle Flexion	Knee Extension	Hip Flexion	Hip Abduction	Hip Adduction	Ankle Extension	Knee Flexion	Hip Extension
	
Primary Torque	Ankle Flexion		X	X					
	Knee Extension	X O		X O	X				
	Hip Flexion		O						
	Hip Abduction							X O	X O
	Hip Adduction						X O	X O	O
	Ankle Extension				X				
	Knee Flexion					X O	X O		X O
	Hip Extension					X O	X O	X O	
	
	Stroke								
	
	Ankle Flexion		X O	X O					
	Knee Extension	X O		X O	X				
	Hip Flexion	O			O				
	Hip Abduction	O	O	X O					
	Hip Adduction								
	Ankle Extension							X	
	Knee Flexion						X O		O
	Hip Extension						O	X O	
	
		Anterior Synergy					Posterior Synergy		

#### Co-contraction index

**Figure 7:**
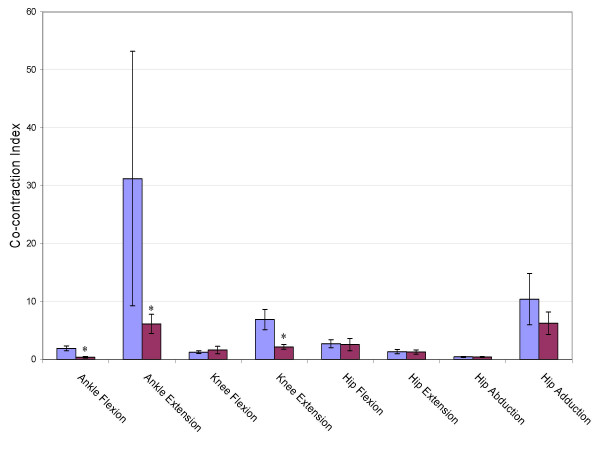
**Co-contraction Index**. Cocontraction index for the eight primary joint torques. Larger values represent lower levels of cocontraction. Error bars represent 95% confidence interval. Significant differences between groups (p < 0.05) are denoted *.

Figure 7 shows the co-contraction index for the eight primary torque directions. The stroke group produced a significantly lower index, and thus greater co-contraction of antagonistic muscle groups during ankle flexion, ankle extension and knee extension. This was especially true during ankle extension where the stroke subjects exerted significantly higher tibialis anterior activity than the control subjects.

## Discussion

### Primary joint torques

As expected, stroke subjects were weaker than age-matched controls for ankle flexion and extension, hip extension, abduction and adduction, and knee flexion. Surprisingly there were no significant differences in hip flexion and knee extension. Even more surprising was that the stroke subjects were, on average, stronger than the control group in knee extension. Median analysis confirms that this is not just the result of a few exceptional stroke subjects. The median stroke knee extension torque was 90.60 Nm while the median control knee extension torque was 81.01 Nm. A closer inspection of the stroke subjects that generated large knee extension or hip flexion torques reveals that these stroke subjects were only stronger in one joint direction, and often generated below average MVT in the other joint directions tested. It is not unreasonable for an ambulatory, active stroke subject to use knee extension as part of a compensatory strategy, and over time, have it be as strong, or stronger, than an age-matched control.

Other factors influencing MVT, such as age, sex, or time post stroke were checked, but no significant correlation was found. However such conclusions are somewhat limited due to our sample size.

### Secondary joint torque patterns

Abnormal coordination patterns in the upper limbs of hemiparetic stroke subjects have been quantified as the generation of torque in joints secondary to the primary joint axis [22]. When this analysis of secondary joint torques was applied to the lower limbs of cerebral palsy subjects, abnormal secondary torques were produced at the hip and knee [23] which were consistent with the classically defined extension synergy [15,16,30]. Presented here is evidence that such classically defined extension and flexion synergy patterns are not present in the lower limbs of chronic stroke subjects while in a functionally relevant standing, weight bearing position.

### Torque patterns of healthy subjects

When asked to generate MVTs along the hip, knee, and ankle flexion and extension axes, the healthy control subjects produced secondary torques in the directions that were consistent with both the mechanical demands of the task and the physical properties of the musculature of the legs. For instance, when asked to generate a maximum knee extension torque, healthy subjects produced secondary hip and ankle flexion torques. So the presence of positive secondary torques of hip and ankle flexion are consistent with mechanical demands of the task. Not surprisingly healthy subjects had a high level of rectus femoris activity during knee extension MVT. The rectus femoris is known as both a knee extensor and hip flexor so the generation of secondary hip flexion during knee extension is consistent with the physical properties of the leg musculature. This led to the grouping of the sagittal plane torques into two synergies. The posterior synergy consisted of hip extension, knee flexion, and ankle extension while the anterior synergy consisted of hip flexion, knee extension, and ankle flexion.

When asked to generate MVTs in the frontal plane joint directions of hip abduction and adduction, healthy subjects produced secondary torques that were not necessarily consistent with the physical properties of the musculature of the legs. The adductor longus is known as a hip flexor as well as adductor, but during high levels of adductor longus activity there was no production of significant hip flexion torque. However, the lower fibers of the gluteus maximus are known to adduct the hip [31] and during high gluteus maximus activity, there were significant secondary hip adduction torques. To further classify the torque patterns of healthy subjects in the frontal plane (joint exertions of hip abduction and adduction) a summary chart of significant secondary torques and correlated joint moments was constructed. Table 2 shows that hip adduction torque was correlated to knee flexion torque (marked 'O'), whereas hip adduction secondary torques were present during knee flexion and hip extension MVTs (marked 'X'). This led to classifying hip adduction as part of the posterior synergy. Even though hip abduction secondary torques were produced during a MVT of an anterior synergy component (knee extension) it has been classified as part of the posterior synergy because hip abduction torque was correlated to knee flexion and hip extension. The presence of hip abduction secondary torques during ankle extension MVT further justifies the posterior synergy classification.

### Torque patterns of chronic stroke subjects

During MVTs in the sagittal plane, chronic stroke subjects showed no evidence of the classic extensor and flexor synergies and behaved similarly to the healthy subjects. The torque patterns of the chronic stroke subjects differed from the healthy subjects only during hip abduction MVT. While healthy subjects produced significant hip extension torques, chronic stroke subjects produced significant hip flexion torque. This abnormal coupling of hip abduction and hip flexion is consistent with the classically defined flexion synergy.

A closer investigation into the secondary torque patterns generated during knee extension revealed that secondary torques were sometimes larger than the torques generated voluntarily. While we cannot conclude this origin for certain, we postulate that a strategy used to generate a MVT may unknowingly involve certain levels of co-contraction that would reduce the net torque. That is, it could be that the agonist muscles may be more active and the antagonistic muscles more relaxed during a strategy used to generate a MVT about a different joint. This would result in a net secondary torque that is larger than a net primary torque. This is not too unusual in the case of chronic stroke subjects generating secondary ankle flexion moments twice as large as their voluntary maximums. The majority of the stroke subjects had poor control at their ankle and often struggled to produce substantial ankle flexion torque. However while concentrating on knee extension exertions, any small increase in a synergistic ankle flexion exertions would be a rather large percentage. The slight increase in tibilias anterior activity from 35.32 % maximum during ankle flexion MVT to 38.78% maximum during knee extension MVT further supports this. Unfortunately this phenomena gets a little more unusual when the levels of co-contraction are compared. A recalculation of ankle co-contraction index during knee extension MVT generation shows that there is a similar amount of co-contraction about the ankle during both voluntary ankle flexion (0.393 +/- 0.279 stdv) and voluntary knee extension (0.396 +/- 0.378 stdv), although recordings of the superficial leg muscles were made. It is likely that had more muscles been recorded from (e.g. soleus) a better understanding for the observed behavior could be explained.

The interesting finding that in control subjects, hip flexion secondary torques were greater than 100% MVT might be explained by the activity of the rectus femoris. During hip flexion MVT control subjects seamed to rely on moderate levels of both rectus femoris (42% maximum) and adductor longus (52% maximum) to achieve hip flexion torques. But during knee extension MVT the rectus femoris activity of the control subjects was higher (54% maximum). A recalculation of hip co-contraction index during knee extension MVT shows that there is less co-contraction about the hip during voluntary knee extension (3.58 +/- 1.39 stdv) than during voluntary hip flexion (2.73 +/- 0.68 stdv). However these findings are not significantly different and had more muscles been recorded from a better understanding for the observed behavior could be explained.

### Weakness in chronic stroke

In a functionally relevant standing position, chronic stroke subjects produced significantly lower torques in six of the eight joint directions tested. Weakness in stroke has been attributed to inadequate recruitment of motorneuron pools [1,4,6] spasticity [10,11], disuse atrophy [3] and the co-contraction of antagonists [7-9]. In an attempt to quantify the amount of co-contraction during the generation of MVTs a co-contraction index was calculated. The chronic stroke subjects produced significantly more co-contraction during ankle flexion and ankle extension which may partially explain the joint torque deficits in those directions. But the stroke subjects produced significantly more co-contraction during knee extension even though they produced a similar level of torque. While inadequate recruitment of motorneuron pools can not be ruled out, it does appear that the co-contraction of antagonistic muscle groups may at least contribute to the observed weakness in the chronic stroke subjects tested. This is consistent with our previous work that demonstrated significant co-activation of antagonistic muscle groups in acute stroke subjects [27].

## Conclusion

Presented here for the first time is a quantitative analysis of lower limb weakness and synergy patterns of chronic stroke subjects in a functionally relevant standing weight-bearing position. In a standing position with added vestibular inputs, stroke subjects showed little evidence of the classic abnormal synergy patterns in seven of the eight directions tested. The findings here suggest that the primary contributor to lower limb motor deficits in chronic stroke subjects is weakness, which is at least partially due to co-contraction of antagonistic muscles.

## Declaration of competing interests

The author(s) declare that they have no competing interests.

## Authors' contributions

NN carried out the experiments, collected and analyzed the data, and drafted the manuscript. MP prepared subjects and assisted with the experiments. DN prepared subjects and assisted with the experiments. JH designed the experiment, developed data collection software, and helped draft the manuscript. All authors read, edited, and approved the final manuscript.
